# Chronic systemic inflammation by peptidoglycan‐polysaccharide induces skeletal muscle atrophy in male and ovariectomized C57BL/6J mice

**DOI:** 10.14814/phy2.70431

**Published:** 2025-06-21

**Authors:** Ryoga Kinoshita, Jay Jung, Sakura Hattori, Tatsuhiro Yamaguchi, Takaya Kotani, Karina Kouzaki, Yuki Tamura, Koichi Nakazato

**Affiliations:** ^1^ Nippon Sport Science University Setagaya Japan; ^2^ The University of Tokyo Meguro Japan; ^3^ Japan Society for the Promotion of Science Chiyoda Japan

**Keywords:** inflammation, sex difference, skeletal muscle

## Abstract

Chronic systemic inflammation (CSI) induces skeletal muscle atrophy. The severity of tissue damage caused by CSI varies according to sex. However, there are still many unknowns regarding the sex differences in skeletal muscle atrophy in patients with CSI. This study aimed to determine the sex differences in CSI‐induced muscle atrophy using male and female C57BL/6J mice. 12‐week‐old mice were divided into peptidoglycan‐polysaccharide (PG‐PS) (each sex, *n* = 9) and control groups (each sex, *n* = 10). In the ovariectomy (OVX) study, 8‐week‐old female C57BL/6J mice were divided into sham, OVX + Saline, and OVX + PG‐PS groups (each group; *n* = 6). A single intraperitoneal injection of PG‐PS or saline was administered to the mice. After 3 weeks, blood and lower leg skeletal muscles were collected. Plasma inflammatory cytokine levels were significantly increased in both sexes following PG‐PS treatment. However, only male mice showed soleus muscle atrophy, decreased muscle protein synthesis (MPS), and increased apoptosis. In the OVX study, the OVX + PG‐PS group exhibited soleus muscle atrophy caused by PG‐PS‐induced CSI. In atrophic muscles, PG‐PS activated p65 and c‐Jun. These findings suggest that ovarian function regulates muscle protein metabolism and suppresses CSI‐induced skeletal muscle atrophy in female mice.

## 1 INTRODUCTION

Chronic systemic inflammation (CSI) is often observed in chronic diseases (e.g., cancer, chronic obstructive pulmonary disease, and chronic kidney disease) and aging (Furman et al., 2019). The inflammatory response is part of the immune defense system activated by several stimulations and infections in the body. However, when immune cells fail to eliminate antigens, acute inflammation develops into chronic inflammation, which is long‐term, low‐grade inflammation (Baylis et al., 2013; Cevenini et al., 2013).

Recent studies have reported that CSI induces skeletal muscle atrophy in males (Doucet et al., 2007; Lee et al., 2017). Skeletal muscle mass depends on the balance between muscle protein synthesis (MPS) and muscle protein breakdown (MPB), and turnover occurs constantly (Burd et al., 2009). However, previous studies have revealed that CSI attenuates MPS and increases apoptosis in the skeletal muscle (Baltgalvis et al., 2010; Beals et al., 2019; Huang et al., 2017). Additionally, various studies have indicated that inflammatory responses differ according to sex. Kosyreva et al. reported that acute systemic inflammation caused more severe tissue and functional disorders in male Wistar rats than in female rats (Kosyreva et al., 2018). Zhong et al. (2022) demonstrated that cancer cachexia‐induced skeletal muscle atrophy was more severe in male mice than in female mice (Zhong et al., 2022). It has been suggested that the sex difference in the inflammatory response is associated with sex hormone levels. The sex hormones primarily produced by the gonads are broadly classified into androgens, estrogens, and progesterone. Androgens are male hormones, including testosterone, dihydrotestosterone, and dehydroepiandrosterone. Androgens suppress immune cell function via androgen receptors (Bevilacqua & Ho, 2022; Lotter et al., 2013). For skeletal muscle, androgens have been shown to increase muscle protein synthesis (Ketchem et al., 2023). In contrast, estrogen is the female hormone comprising oestradiol, oestrone, and estriol. It activates immune cells via estrogen receptors (Kumwenda et al., 2022; Leone et al., 2004). Additionally, estrogen protects myocytes from several stressors, such as inflammation and reactive oxygen species (ROS) (Karvinen et al., 2021; Xu et al., 2018). Progesterone has been reported to suppress the activity of immune cells and promote muscle protein synthesis in skeletal muscles (Gay et al., 2021; Toth et al., 2001). However, the mechanisms underlying sex differences in CSI‐induced skeletal muscle atrophy remain unclear because of the complexity of the immune system and disease diversity. Therefore, understanding how CSI affects skeletal muscle atrophy is crucial for improving health outcomes.

Various chemical components can induce systemic inflammation in animals. Lipopolysaccharide (LPS), a component of gram‐negative bacteria, is commonly used (Kawamura et al., 2019; Liu et al., 2014; Zhao et al., 2016). However, a previous study reported that it caused severe inflammation and organ failure, such as sepsis (Watts et al., 2020). Thus, the induction of chronic inflammation by LPS requires frequent administration of small doses, making chronic intervention difficult. In contrast, peptidoglycan‐polysaccharide (PG‐PS), a gram‐positive bacterial component found in *Staphylococcus* and *Streptococcus*, induces long‐term inflammation without causing death after a single dose (Garcia et al., 2011; Hannig et al., 2007; Kimpel et al., 2003; Theurl et al., 2009). Recently, Sumi et al. reported that a single PG‐PS administration (5 μg/g body weight [BW]) induced CSI, skeletal muscle atrophy, and liver dysfunction in female Lewis rats (Sumi et al., 2020b). PG‐PS has the advantage of causing CSI‐induced skeletal muscle atrophy more easily than LPS. This study aimed to determine whether PG‐PS administration‐induced skeletal muscle atrophy and its underlying mechanisms differ by sex. Additionally, we hypothesized that ovarian function is involved in sex differences in muscle atrophy and examined the effects of PG‐PS administration in OVX mice.

## 2 METHODS

### 2.1 Materials

The 10S fraction of PG‐PS was purchased from Becton Dickinson and Company (210866; Franklin Lakes, NJ, USA).

### 2.2 Animals and experimental protocols

All animal experiments were approved by the Animal Experimental Committee of Nippon Sport Science University (approval no. 021‐A03). Twelve‐week‐old male and female C57BL/6J mice were used in this study (CREA Japan, Tokyo, Japan). Mice were housed individually in an animal cage maintained at 23°C with a 12:12 h light–dark cycle. They were fed a standard rodent solid diet (CE‐2; CREA Japan) and had ad libitum access to water for at least 1 week to acclimate.

After 1 week of acclimatization, the mice were divided into PG‐PS (each sex; *n* = 9) and control groups (each sex; *n* = 10). A single intraperitoneal injection of PG‐PS or saline (50 μg/g BW) was administered to the mice. BW and food intake were measured every 3 days. Three weeks after PG‐PS administration, the mice were fasted overnight, and blood was collected from the tail vein. Subsequently, cervical dislocation was carefully performed to minimize any potential pain or distress, followed by dissection. We measured the wet weights of the lower hindlimb muscles (gastrocnemius, plantaris, and soleus muscles). Cross‐sectional area (CSA) samples were embedded in Tissue‐Tek O.C.T Compound (4583, Sakura Finetek, Tokyo, Japan) and stored at −80°C. Other tissues were rapidly frozen in liquid nitrogen within 15 min of cervical dislocation and stored at −80°C until further analyses.

### 2.3 Cytokine measurement

Inflammatory cytokine levels in tail vein‐derived plasma were measured using an ELISA kit according to the manufacturer's protocols (TNF‐α: MTA00B; IL‐1β: MLB00C; R&D Systems, Minneapolis, MN).

### 2.4 Determination of muscle fiber size and fiber type

Immunohistochemistry for the different myosin heavy chain isoforms was performed as described previously (Yamaguchi et al., 2024). Cross‐sections of the soleus muscle (10 μm‐thick) were prepared using a cryostat (CM1860; Leica Biosystems, Nussloch, Germany) at −20°C. Subsequently, the sections were blocked with 4% bovine serum albumin (BSA) blocking buffer (015‐21274; Fujifilm Wako Pure Chemical Corporation, Osaka, Japan) for 1 h at room temperature. The sections were then incubated overnight at 4°C with the following primary antibodies diluted in BSA blocking buffer: rabbit immunoglobulin G (IgG) polyclonal anti‐laminin antibody (1:1000 dilution; L9393; Sigma‐Aldrich, St. Louis, MO, USA), mouse IgG2b polyclonal anti‐myosin heavy chain type I (1:500 dilution; BA‐D5‐s; DSHB, Iowa, USA), and IgG1 polyclonal anti‐myosin heavy chain type IIa (1:500 dilution; SC‐71‐s; DSHB). After incubation, the sections were washed three times for 5 min each in 0.1 M phosphate buffer (PB; pH 7.4). The sections were incubated overnight in the dark at 4°C with the following secondary antibodies diluted in BSA blocking buffer: Alexa Fluor™ Plus 647 goat anti‐rabbit IgG H+L (1:1000 dilution; A32733; Invitrogen, Waltham, MA, USA), Alexa Fluor 555 goat anti‐mouse IgG2b (1:1000 dilution; A21147; Invitrogen), and Alexa Fluor 488 goat anti‐mouse IgG1 (1:1000 dilution; A21121; Invitrogen). Following incubation, the sections were washed again in 0.1 M PB. The sections were mounted on slides with coverslips using the Fluoro‐KEEPER Antifade reagent (12593‐64; Nacalai Tesque, Kyoto, Japan). The slides were imaged using a confocal microscope (FV3000; Olympus, Tokyo, Japan). The myofibre size and fiber‐type distribution were analyzed using MyoVision software, according to a previously described method (Wen et al., 2018). The average number of muscle fibers per group was as follows: male: 301 ± 34 (control), 339 ± 45 (PG‐PS); female: 325 ± 50 (control), 339 ± 20 (PG‐PS); OVX: 374 ± 63 (sham), 357 ± 75 (OVX + Saline), 408 ± 39 (OVX + PG‐PS).

### 2.5 Western blotting analysis

The protein detection by western blotting was conducted following the methodology described in a previous study (Kasai et al., 2022). The gastrocnemius and soleus muscles were homogenized in radioimmunoprecipitation buffer (188‐02453, Fujifilm Wako Pure Chemical Corporation, Osaka, Japan) containing protease and phosphate inhibitor cocktails (169‐26063/167‐24381, Fujifilm Wako Pure Chemical Corporation, Osaka, Japan), respectively. The homogenates were centrifuged at 15,000×*g* for 15 min at 4°C, and the supernatants were collected. The protein concentrations in the lysates were measured using a bicinchoninic acid (BCA) protein assay kit (23225, Pierce BCA Protein Assay Kit; Thermo Fisher Scientific, Waltham, MA, USA). Equal amounts (5 μg) of proteins were separated via sodium dodecyl sulfate‐polyacrylamide gel electrophoresis (10% [wt/vol]; TGX polyacrylamide gel, 161–0173, Bio‐Rad, CA, USA) and transferred to polyvinylidene difluoride membranes (IPVH00010, Immobilon‐P Membrane; EMD Millipore, Billerica, MA, USA). Protein transfer was confirmed by Ponceau S staining (33427.01; SERVA Electrophoresis, Heidelberg, Germany). The membranes were blocked with a blocking reagent (NYPBR01; Toyobo Company) for 1 h at 25°C and then incubated overnight with primary antibodies diluted in Reagent 1 (NKB‐101; Toyobo Company). The following antibodies were used: anti‐puromycin (MABE343; EDM Millipore) phospho p65 (3033; Cell Signaling Technology [CST], Danvers, MA, USA), total p65 (8242; CST); phospho c‐Jun (2361; CST), total c‐Jun (9165; CST), Bim (2933; CST), and Bax (2772; CST). All primary antibodies were diluted 1:1000. After incubation, the membranes were washed three times for 5 min each in Tris‐buffered saline containing 0.01% Tween 20 (TBST; T9142; Takara Bio, Shiga, Japan). They were then incubated for 1 h at 25°C with secondary antibodies (anti‐rabbit or anti‐mouse IgG HRP‐linked antibody; 7074/7076, CST, Danvers, MA, USA) diluted in Reagent 2 (NKB‐101; Toyobo Company). After incubation, the membranes were washed again with TBST. Specific protein bands were visualized using a chemiluminescent reagent (34579; Super Signal West Pico Chemiluminescent Substrate; Thermo Fisher Scientific). The blots were scanned using a CCD imager (170‐8071, ChemiDoc XRS; Bio‐Rad) and quantified using Quantity One (170‐9600, for Windows, v.5.2.1, Bio‐Rad). Ponceau S signal intensity was used as the loading control.

### 2.6 Terminal dUTP nick‐end labeling (TUNEL) staining

The apoptotic nuclei in the muscle fibers were assessed using a TUNEL kit (11684795910; Roche Applied Science, Mannheim, Germany). Briefly, soleus muscle sections (10 μm‐thick) were prepared using a cryostat and dried at 25°C. The sections were fixed with 4% paraformaldehyde in phosphate‐buffered saline (PBS; 006775; Bioenno Tech, California, USA) at 25°C for 60 min. After fixation, the cells were permeabilised with 0.1% Triton X‐100 and 0.1% sodium citrate in PBS at 4°C for 2 min. Subsequently, the tissues were incubated with a TUNEL reaction mixture at 37°C in a humidified chamber for 60 min. Following labelling, tissue sections were incubated with primary rabbit IgG polyclonal anti‐laminin antibody diluted in BSA blocking buffer overnight at 4°C. After incubation, the sections were washed in 0.1 M PB and incubated with a secondary antibody, Alexa Fluor 555 goat anti‐rabbit IgG2b (1:1000 dilution; A21428; Invitrogen) for 60 min in the dark. To stain nuclei, the sections were mounted with 4′,6‐diamidino‐2‐phenylindole (DAPI; 1274574; Nacalai Tesque). The nuclei were imaged using a confocal laser scanning microscope (FV3000; Olympus). DAPI‐ and TUNEL‐positive nuclei were quantified, and the data were expressed as the TUNEL index (TUNEL‐positive nuclei/DAPI‐positive nuclei × 100).

### 2.7 MPS

MPS was assessed using the in vivo SUnSET method in half of the mice of each sex (male; Con: *n* = 5, PG: *n* = 5, female; Con: *n* = 5, PG: *n* = 4) (Goodman et al., 2011). Puromycin (0.04 μmol/g BW in saline) (160‐23151; Fujifilm Wako Pure Chemical Corporation, Osaka, Japan) was intraperitoneally injected into mice under isoflurane anesthesia to measure MPS. After the injection, skeletal muscles and adipose tissues were collected 15 min later. As described above, puromycin incorporation into the gastrocnemius muscle was detected via western blotting analysis.

### 2.8 Ovariectomy (OVX) mouse study

Eight‐week‐old female C57Bl/6J mice were randomly divided into control (sham), OVX + Saline, and OVX + PG‐PS groups. After acclimation, OVX was performed through a dorsal incision under 1.6% isoflurane anesthesia on mice in the OVX + Saline and OVX + PG‐PS groups. The sham group underwent the same surgical procedures as the others, except for the OVX procedure (Anesthesia, incisions, sutures, etc.). Four weeks after OVX, mice were intraperitoneally injected with saline or PG‐PS (50 μg/g BW). On day 21, puromycin (0.04 μmol/g BW in saline) was intraperitoneally injected under isoflurane anesthesia after fasting overnight. After 15 min, skeletal muscle was dissected and collected.

### 2.9 Statistical Analysis

All values are presented as the mean ± standard deviation (SD). Student's unpaired *t*‐test was used to assess differences between the control and PG‐PS groups. The effects of time, group, or their interaction were tested using two‐way analysis of variance (ANOVA) and Tukey's post hoc test. For the OVX study, data were analyzed using one‐way and two‐way ANOVAs, followed by Tukey's post hoc test. Statistical significance was set at *p* < 0.05. All the analyses were performed using GraphPad Prism 9 (for Mac, version 9.4.0; GraphPad Software, CA, USA).

## 3 RESULTS

### 3.1 PG‐PS Administration‐induced CSI in both sexes

**FIGURE 1:**
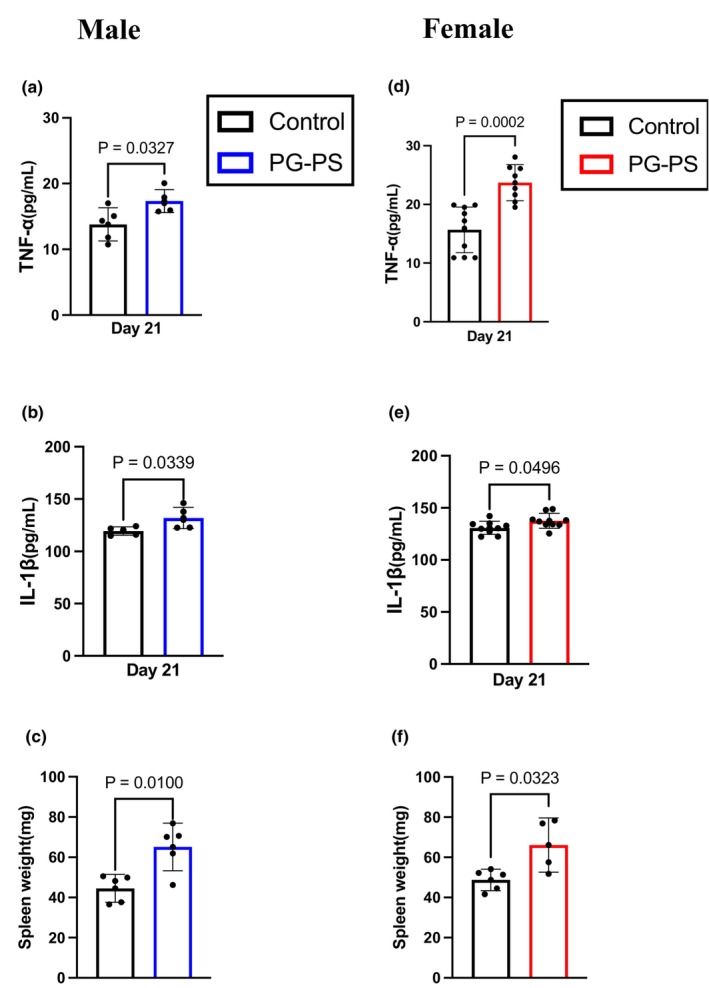
Inflammatory cytokine level and spleen weight in Saline and PG‐PS mice. (a) Plasma concentration of TNF‐α in male mice (Con: *n* = 5, PG: *n* = 5). (b) Plasma concentration of IL‐1β in male mice (Con: *n* = 5, PG: *n* = 5). (c) Plasma concentration of TNF‐α in female mice (Con: *n* = 10, PG: *n* = 9). (d) Plasma concentration of IL‐1β in female mice (Con: *n* = 9, PG *n* = 9). (e) Spleen weight of male mice (Con: *n* = 5, PG: *n* = 5). (f) Spleen weight of female mice (Con: *n* = 5, PG: *n* = 4). Data are shown as the mean ± SD. Student's unpaired *t*‐test was performed.

First, we determined whether PG‐PS induces CSI in mice based on the plasma levels of TNF‐α and IL‐1β. Three weeks after the injection, the average concentrations of TNF‐α and IL‐1β were significantly higher in the PG‐PS‐treated group than those in the control groups for both sexes (*p* = 0.032, *p* = 0.033, *p* < 0.001, and *p* = 0.049, respectively, Figure 1a,b,d,e). Additionally, the spleen, which is involved in systemic inflammation, significantly increased in weight in both sexes following PG‐PS administration (*p* = 0.01 and *p* = 0.032, respectively, Figure 1c,f). This indicates that PG‐PS induced CSI in both sexes.

### 3.2 PG‐PS decreased BW and food intake in male mice

**FIGURE 2:**
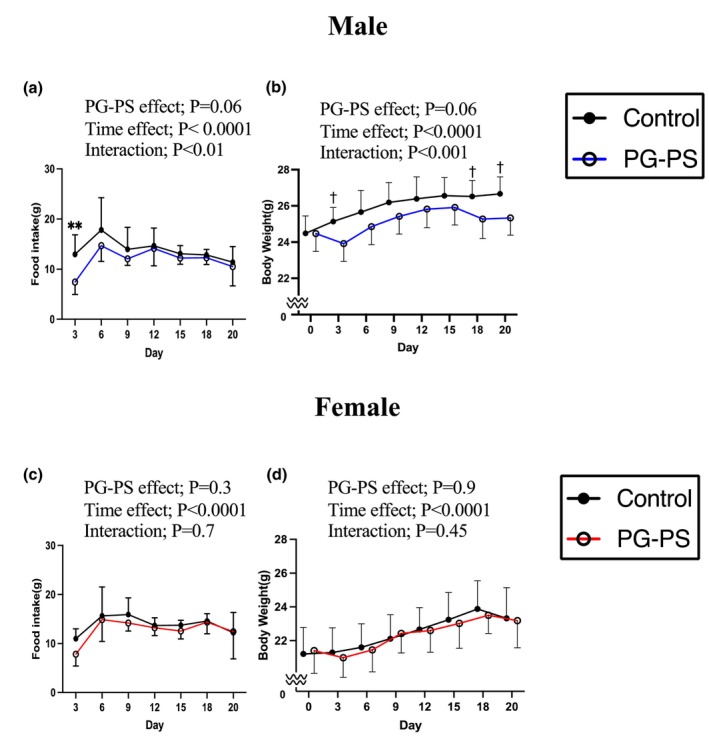
Body weight and food intake. (a) Food intake of male mice (Con: *n* = 10, PG: *n* = 9). (b) Body weight of male mice (Con: *n* = 10, PG: *n* = 9). (c) Food intake of female mice (Con: *n* = 10, PG: *n* = 9). (d) Body weight of female mice (Con: *n* = 10, PG: *n* = 9). Data are shown as the mean ± SD. Two‐way analysis of variance (ANOVA) was performed, followed by a post hoc test. ^†^ *p* < 0.1, **p < 0.01.

We next examined BW and food intake every 3 days after the PG‐PS injection. Food intake in male PG‐PS mice was lower than in the control group on Day 3, whereas female mice showed no significant differences between groups (*p* = 0.002 and *p* > 0.999, respectively, Figure 2a,c). The BWs of male mice were lower in the PG‐PS group on days 3, 18, and 20 (*p* = 0.076, *p* = 0.066, and *p* = 0.056, respectively, Figure 2b). However, no significant differences in BW were observed in female mice (Figure 2d). These findings indicate that PG‐PS administration affected BW and food intake only in male mice.

### 3.3 PG‐PS Administration reduced white adipose tissue and skeletal muscle mass in male mice

**FIGURE 3:**
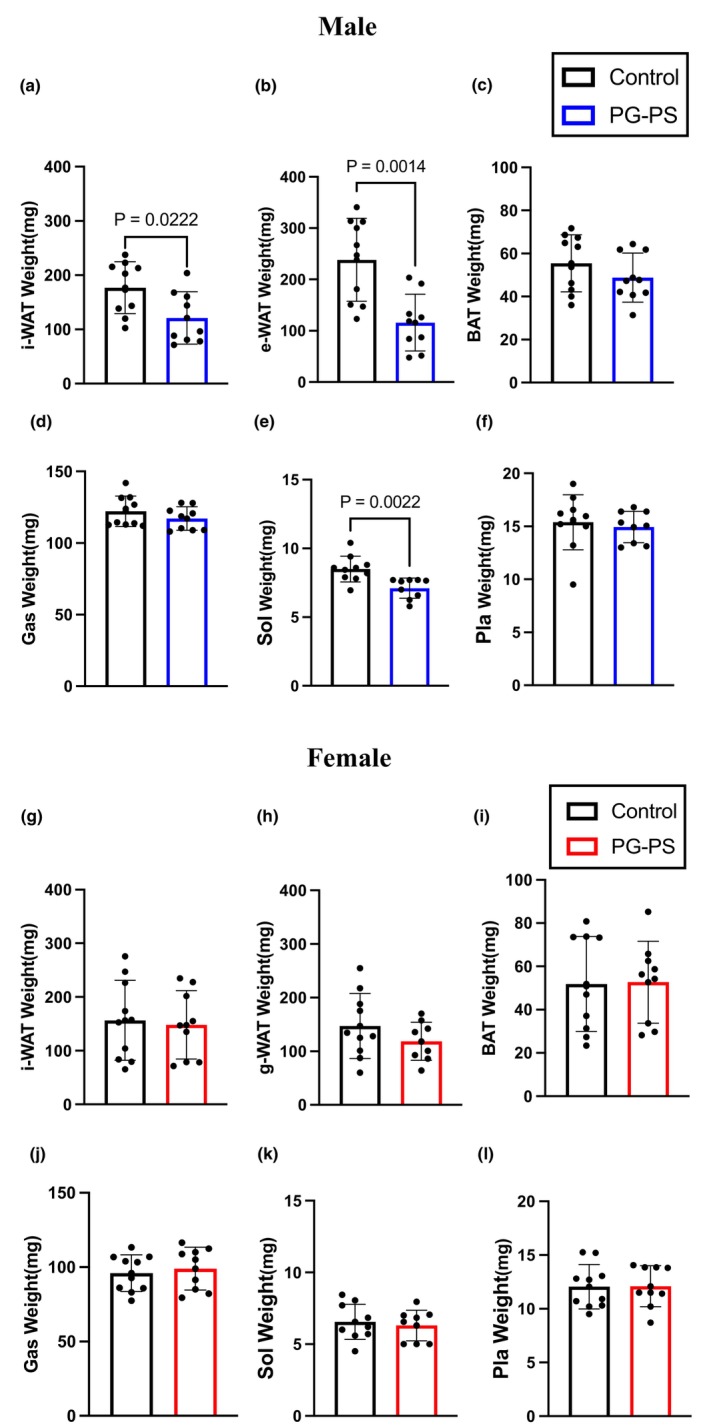
Adipose tissue and skeletal muscle weight. (a) Inguinal white adipose tissue weight of male mice (Con: *n* = 10, PG: *n* = 9). (b) Epididymis white adipose tissue weight of male mice (Con: *n* = 10, PG: *n* = 9). (c) Neck brown adipose tissue weight of male mice (Con: *n* = 10, PG: *N* = 9). (d) Gastrocnemius muscle weight of male mice (Con: *n* = 10, PG: *N* = 9). (e) Soleus muscle weight of male mice (Con: *n* = 10, PG: *n* = 9). (f) Plantaris muscle weight of male mice (Con: *n* = 10, PG: *n* = 9). (g) Inguinal white adipose tissue weight of female mice (Con: *n* = 10, PG: *n* = 9). (h) Gonadal fat white adipose tissue weight of female mice (Con: *n* = 10, PG: *n* = 9). (i) Neck brown adipose tissue weight of female mice (Con: *n* = 10, PG: *n* = 9). (j) Gastrocnemius muscle weight of female mice (Con: *n* = 10, PG: *n* = 9). (k) Soleus muscle weight of female mice (Con: *n* = 10, PG: *n* = 9). (l) Plantaris muscle weight of female mice (Con: *n* = 10, PG: *n* = 9). Data are shown as the mean ± SD. Two‐way analysis of variance (ANOVA) was performed, followed by a post hoc test. A comparison of adipose and skeletal muscle weight was used in a student's unpaired *t*‐test.

In this study, a reduction in white adipose tissue was observed in male mice following PG‐PS administration (*p* = 0.022, *p* = 0.001, and *p* = 0.258, respectively, Figure 3a–c). Still, it was not evident in female mice (*p* = 0.785, *p* = 0.234, and *p* = 0.929, respectively, Figure 3g–i). This likely contributed to the body weight loss observed in male mice.

Next, we measured the wet weights of the gastrocnemius, plantaris, and soleus muscles. In the present study, a significant decrease in soleus muscle weight after PG‐PS administration was observed in male mice (*p* = 0.262, *p* = 0.649, and *p* = 0.002, respectively, Figure 3d–f). Conversely, no significant differences in skeletal muscle weight were observed between PG‐PS‐treated and control groups in female mice (*p* = 0.625, *p* = 0.970, and *p* = 0.630, respectively, Figure 3j–l). These results indicated there is a sex difference in PG‐PS‐induced muscle atrophy, which was more remarkable in male than in female mice.

### 3.4 PG‐PS decreased the CSA of the soleus muscle in male mice

**FIGURE 4:**
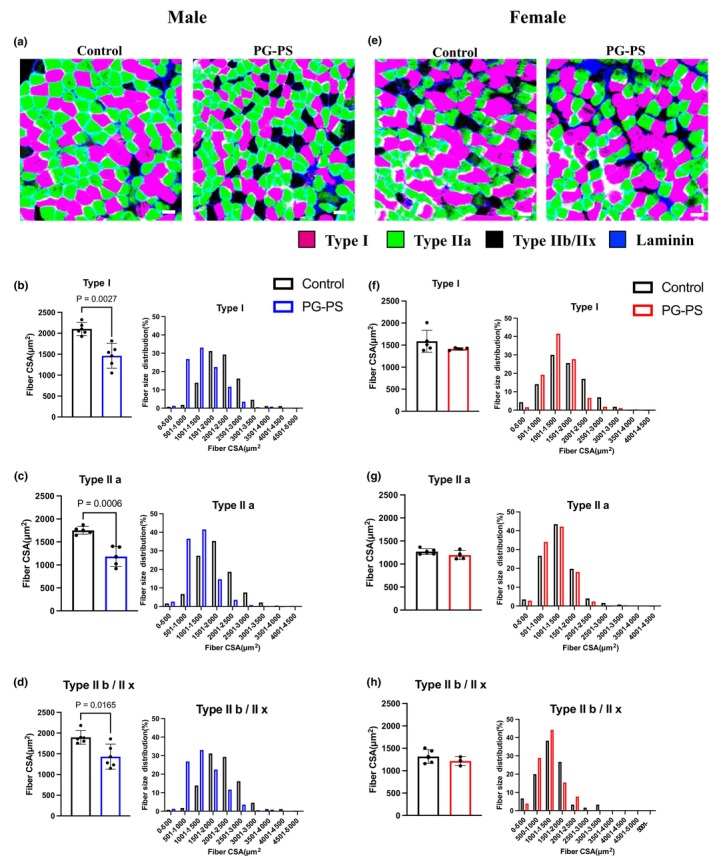
Muscle fiber CSA in soleus muscle each sex. (a) Representative image of soleus muscle in male mice (Scale bar = 100 μm). (b) Type I fiber CSA and fiber size distribution of soleus muscle in male mice (Con: *n* = 5, PG: *n* = 5). (c) Type IIa fiber CSA and fiber size distribution of soleus muscle in male mice (Con: *n* = 5, PG: *n* = 5). (d) Type IIb/IIx fiber CSA and fiber size distribution of soleus muscle in male mice (Con: *n* = 5, PG: *n* = 5). (e) Representative image of soleus muscle in female mice (Scale bar = 100 μm). (f) Type I fiber CSA and fiber size distribution of soleus muscle in female mice (Con: *n* = 5, PG: *n* = 4). (g) Type IIa fiber CSA and fiber size distribution of soleus muscle in female mice (Con: *n* = 5, PG: *n* = 4). (h) Type IIb/IIx fiber CSA and fiber size distribution of soleus muscle in female mice (Con: *n* = 5, PG: *n* = 4). Data are shown as the mean ± SD. Student's unpaired *t*‐test was performed.

CSA of the soleus muscle fibers was measured to analyze the details of muscle atrophy. The size of type I, IIa, IIx/IIb fibers was significantly reduced with PG‐PS treatment in male mice (*p* = 0.002, *p* < 0.001, and *p* = 0.016, respectively, Figure 4a–d). Conversely, no significant differences in the CSAs were observed between the PG‐PS and control groups in female mice across each type of fiber (*p* = 0.22, *p* = 0.206, and *p* = 0.357, respectively, Figure 4e–h).

### 3.5 PG‐PS activated apoptosis in the male soleus muscle

**FIGURE 5:**
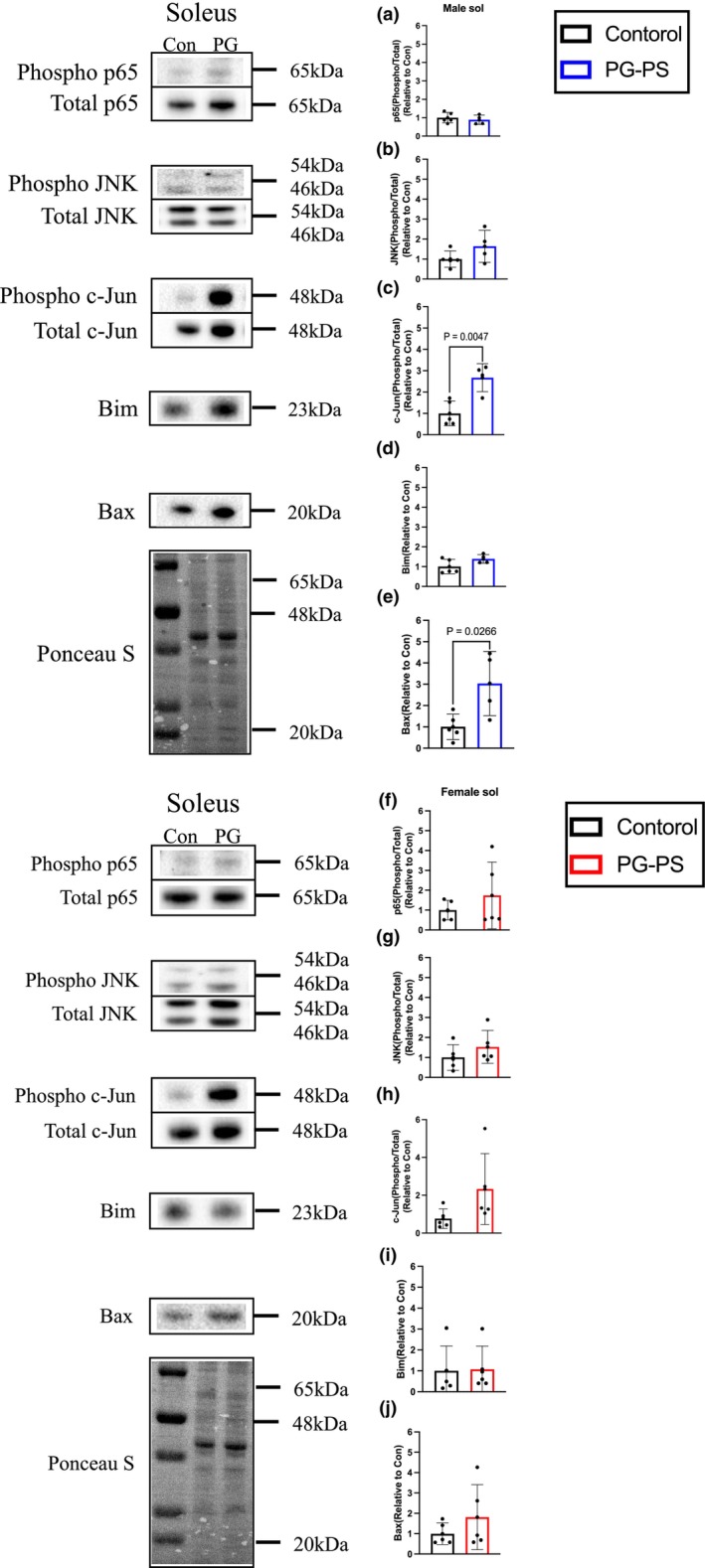
Inflammation‐related proteins in each sex. (a) Phosphorylated per total p65 of soleus muscle in male mice (Con: *n* = 5, PG: *n* = 4). (b) Phosphorylated per total JNK of soleus muscle in male mice (Con: *n* = 5, PG: *n* = 4). (c) Phosphorylated per total c‐Jun of soleus muscle in male mice (Con: *n* = 5, PG: *n* = 4). (d) Expression of Bim in male mice soleus muscle (Con: *n* = 5, PG: *n* = 4). (e) Expression of Bax in male mice soleus muscle (Con: *n* = 5, PG: *n* = 4). (f) Phosphorylated per total p65 of soleus muscle in female mice (Con: *n* = 5, PG: *n* = 5). (g) Phosphorylated per total JNK of soleus muscle in female mice (Con: *n* = 5, PG: *n* = 5). (h) Phosphorylated per total c‐Jun of soleus muscle in female mice (Con: *n* = 5, PG: *n* = 5). (i) Expression of Bim in female mice soleus muscle (Con: *n* = 5, PG: *n* = 5). (j) Expression of Bax in female mice soleus muscle (Con: *n* = 5, PG: *n* = 5). Data are shown as the mean ± SD. Students' unpaired *t*‐test was performed.

**FIGURE 6:**
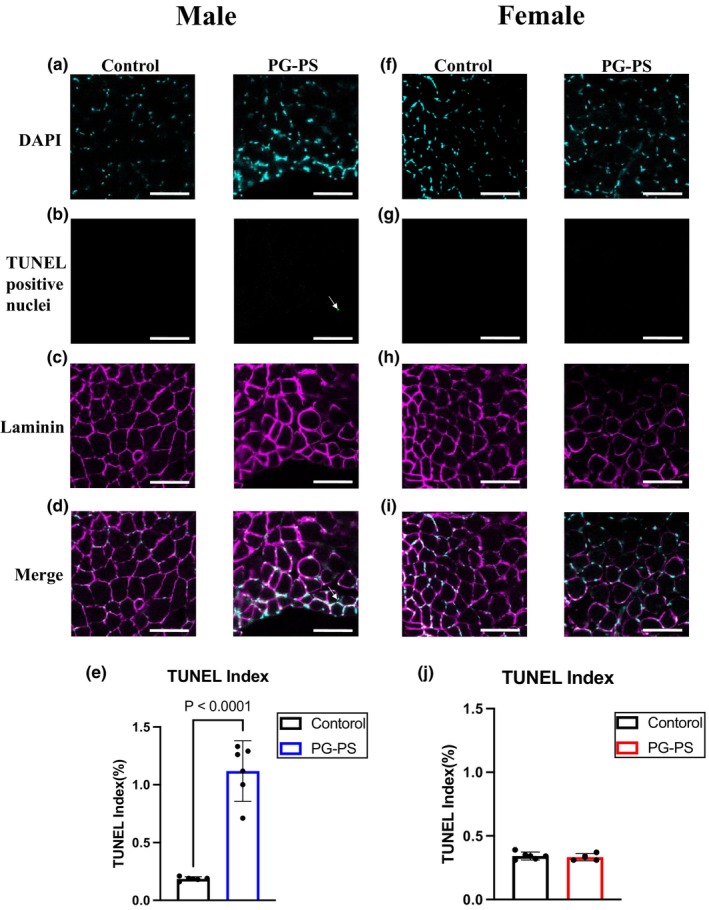
Terminal dUTP nick‐end labelling (TUNEL) and 4′6, −diamidno‐20phenylindole (DAPI) staining for control and PG‐PS. (a) DAPI of soleus muscle in male mice (Scale bar = 100 μm). (b) TUNEL‐positive nuclei of soleus muscle in male mice (Scale bar = 100 μm). (c) Laminin of soleus muscle in male mice (Scale bar = 100 μm). (d) Merge image of soleus muscle in male mice (Scale bar = 100 μm). (e) TUNEL index in male mice (Con: *n* = 5, PG: *n* = 5). (f) DAPI of soleus muscle in female mice. (g) TUNEL‐positive nuclei of soleus muscle in female mice (Scale bar = 100 μm). (h) Laminin of soleus muscle in female mice (Scale bar = 100 μm). (i) Merge image of soleus muscle in female mice (Scale bar = 100 μm). (j) TUNEL index in female mice (Con: *n* = 5, PG: *n* = 4). Data are shown as the mean ± SD. Student's unpaired *t*‐test was performed.

Next, we used western blotting to measure proteins associated with TNF signaling. In both sexes, phosphorylated p65 levels in the soleus muscle did not differ significantly between PG‐PS and control groups. However, in male mice, the levels of activated c‐Jun and Bax in the PG‐PS group were considerably higher than those in the control (*p* = 0.553, *p* = 0.161, *p* = 0.004, *p* = 0.101, and *p* = 0.026, respectively, Figure 5a–e). Conversely, no significant differences were observed in female mice between groups (*p* = 0.371, *p* = 0.289, *p* = 0.111, *p* = 0.923, and *p* = 0.311, respectively, Figure 5f–j). These results suggest that the activation of apoptosis decreases the skeletal muscle mass of PG‐PS‐treated male mice. Next, we performed TUNEL staining to observe apoptosis in the soleus muscle. TUNEL‐positive nuclei were rarely observed in the soleus muscle of male control mice. However, PG‐PS treatment significantly increased the TUNEL index in male mice. In contrast, no significant differences in the TUNEL index were observed between the control and PG‐PS groups in female mice (*p* < 0.0001 and *p* = 0.652, respectively, Figure 6). These results are consistent with the activation of apoptosis‐related proteins.

### 3.6 PG‐PS administration reduced MPS in male mice

**FIGURE 7:**
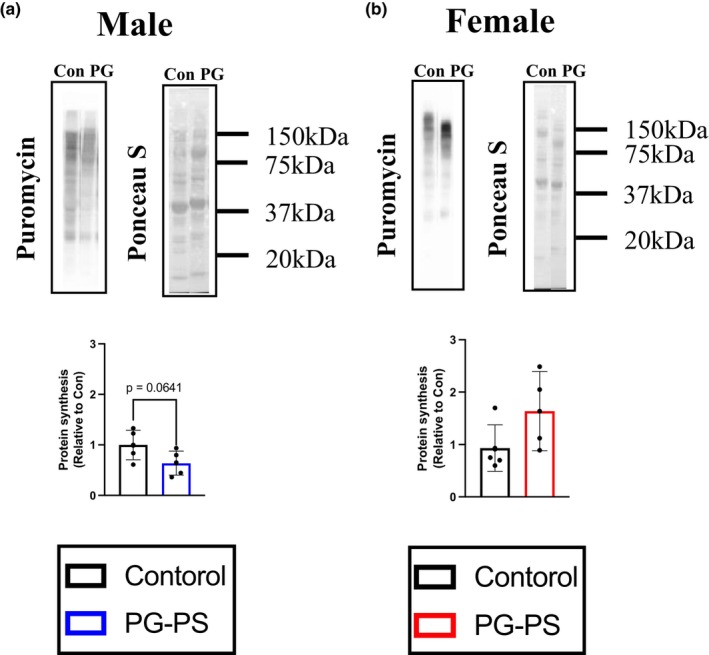
MPS in each sex. (a) Puromycin‐incorporated protein and Ponceau S of gastrocnemius muscle in male mice. (Con: *n* = 5, PG: *n* = 5). (b) Puromycin‐incorporated protein and Ponceau S of gastrocnemius muscle in female mice. (Con: *n* = 5, PG: *n* = 4). Data are shown as the mean ± SD. Student's unpaired *t*‐test was performed.

We subsequently investigated how PG‐PS‐induced CSI affects MPS. PG‐PS administration decreased baseline MPS in male mice, whereas no significant differences were observed in female mice between the control and PG‐PS groups (*p* = 0.064 and *p* = 0.122, respectively, Figure 7a,b).

### 3.7 Effects of CSI on OVX mice

The previously observed sex differences in PG‐PS‐induced skeletal muscle atrophy may be attributed to the effects of sex hormones. To investigate this, we assessed the impact of OVX on skeletal muscle atrophy after PG‐PS administration.

**FIGURE 8:**
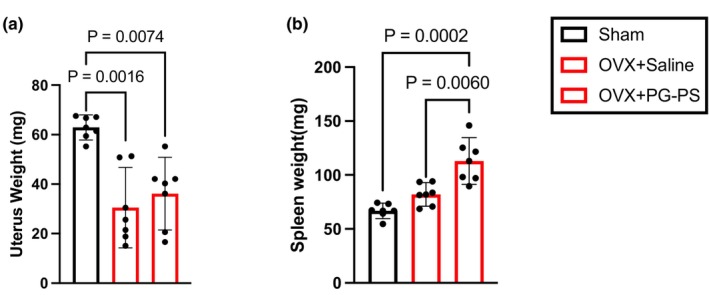
Uterus and spleen weight in the OVX study. (a) Uterus weight (Sham: *n* = 6, OVX + Saline; *n* = 6, OVX + PG‐PS: *n* = 6). (b) Spleen weight (Sham: *n* = 6, OVX + Saline; *n* = 6, OVX + PG‐PS: *n* = 6). Data are shown as the mean ± SD. One‐way analysis of variance (ANOVA) was performed, followed by a post hoc test.

Menopause and OVX induce uterine atrophy (Elliot et al., 2003; Mann et al., 2020) in mice. In this study, the uterine weights of the mice in the OVX groups were lower than those in the sham group, indicating that the OVX surgery was conducted appropriately (*p* = 0.001 and *p* = 0.007, Figure 8a). In addition, the spleen weights in the OVX + PG‐PS group were higher than those in the sham and OVX + Saline groups (*p* < 0.001 and *p* = 0.006, respectively, Figure 8b), suggesting that PG‐PS induced CSI even in OVX mice.

### 3.8 PG‐PS administration‐induced skeletal muscle atrophy in OVX mice

**FIGURE 9:**
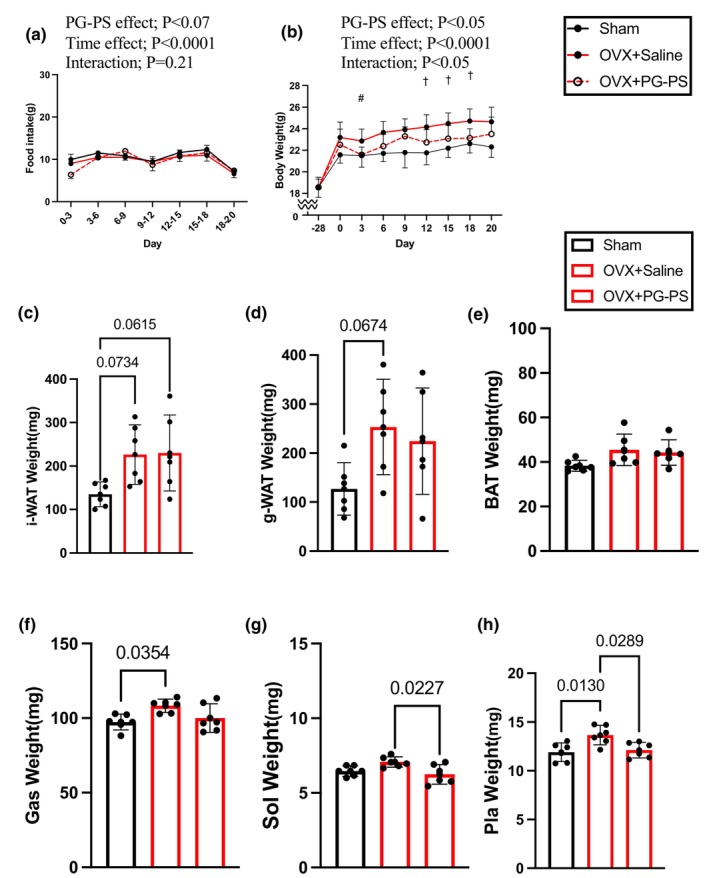
Body weight, food intake, adipose tissue, and skeletal muscle weight of OVX mice. (a) Food intake (Sham: *n* = 6, OVX + Saline; *n* = 6, OVX + PG‐PS: *n* = 6). (b) Body weight (Sham: *n* = 6, OVX + Saline; *n* = 6, OVX + PG‐PS: *n* = 6). (c) Inguinal white adipose tissue weight (Sham: *n* = 6, OVX + Saline; *n* = 6, OVX + PG‐PS: *n* = 6) (d) Gonadal fat white adipose tissue weight (Sham: *n* = 6, OVX + Saline; *n* = 6, OVX + PG‐PS: *n* = 6) (e) Neck brown adipose tissue weight (Sham: *n* = 6, OVX + Saline; *n* = 6, OVX + PG‐PS: *N* = 6) (f) Gastrocnemius muscle weight (Sham: *n* = 6, OVX + Saline; *n* = 6, OVX + PG‐PS: *n* = 6). (g) Soleus muscle weight (Sham: *n* = 6, OVX + Saline; *n* = 6, OVX + PG‐PS: *n* = 6). (h) Plantaris muscle weight (Sham: *n* = 6, OVX + Saline; *n* = 6, OVX + PG‐PS: *n* = 6). Data are shown as the mean ± SD. One‐way analysis of variance (ANOVA) was performed, followed by a post hoc test. Body weight data: *p < 0.05 (Sham vs. OVX + Saline), ^#^ *p* < 0.1 (OVX + Saline vs. OVX + PG‐PS), ^†^ *p* < 0.05 (OVX + Saline vs. OVX + PG‐PS); Skeletal muscle: *p < 0.05.

Food intake did not differ among the sham, OVX + Saline, and OVX + PG‐PS groups during the intervention (Figure 9a). However, the weight of the OVX + Saline group increased significantly compared to that of the sham group at all time points. In contrast, the BW in the OVX + PG‐PS group was lower than that in the OVX + Saline group on Days 12, 15, and 18 (*p* = 0.008–0.042, Figure 9b). This indicates that PG‐PS‐induced CSI decreases BW in OVX mice, similar to its effects in male mice.

Weight gain in OVX mice is associated with increased fat mass. To investigate this, white and brown adipose tissue samples were collected and weighed. White adipose tissue weight was generally higher in OVX rats than in the sham group (*p* = 0.073, *p* = 0.061, *p* = 0.067, *p* = 0.178, *p* = 0.091, and *p* = 0.178 respectively, Figure 9c–e). However, there was no difference among the OVX groups (*p* = 0.995, *p* = 0.844, and *p* = 0.919, respectively, Figure 9c–e).

Next, we examined whether PG‐PS induces skeletal muscle atrophy in OVX mice. A previous study reported that after surgery, OVX mice had temporarily increased skeletal muscle mass. OVX surgery significantly increased the weight of gastrocnemius and plantaris muscles. However, soleus and plantaris muscle levels in the OVX + PG‐PS group were considerably lower than those in the OVX + Saline group (*p* = 0.022 and *p* = 0.028, respectively, Figure 9f–h).

### 3.9 PG‐PS treatment decreased the CSA of the soleus muscle in OVX mice

**FIGURE 10:**
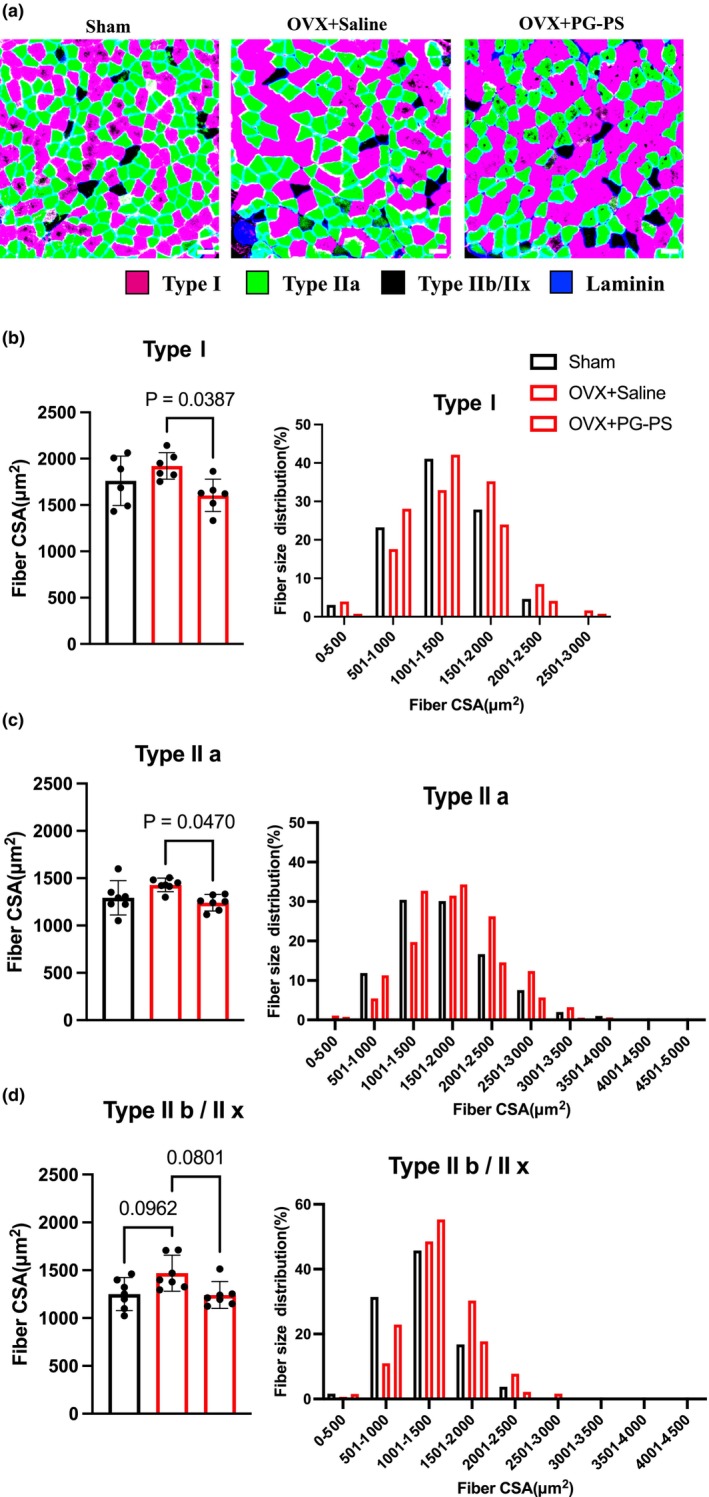
Muscle fiber CSA of soleus muscle in OVX mice. (a) Representative image of soleus muscle in female mice (Scale bar = 100 μm). (b) Type I fiber CSA and fiber size distribution of soleus muscle in female mice (Sham: *n* = 6, OVX + Saline; *n* = 6, OVX + PG‐PS: *n* = 6). (c) Type IIa fiber CSA and fiber size distribution of soleus muscle in female mice (Sham: *n* = 6, OVX + Saline; *n* = 6, OVX + PG‐PS: *n* = 6). (d) Type IIb/IIx fiber CSA and fiber size distribution of soleus muscle in female mice (Sham: *n* = 6, OVX + Saline; *n* = 6, OVX + PG‐PS: *n* = 6). Data are shown as the mean ± SD. One‐way analysis of variance (ANOVA) was performed, followed by a post hoc test.

As described earlier, PG‐PS administration reduces skeletal muscle weight in OVX mice. To further investigate this, we measured the CSA of the skeletal muscle fibers. In the soleus muscle, the CSA of Type IIb/x fibers tended to increase in OVX mice compared to those in the sham group. However, PG‐PS administration reduced the CSA of all muscle fiber types in the OVX + PG‐PS group compared to that in the OVX + Saline group (*p* = 0.038, *p* = 0.047, and *p* = 0.080, respectively, Figure 10). These results indicate that PG‐PS induces skeletal muscle atrophy in OVX mice.

### 3.10 PG‐PS Administration increased inflammation‐related proteins in OVX mice

**FIGURE 11:**
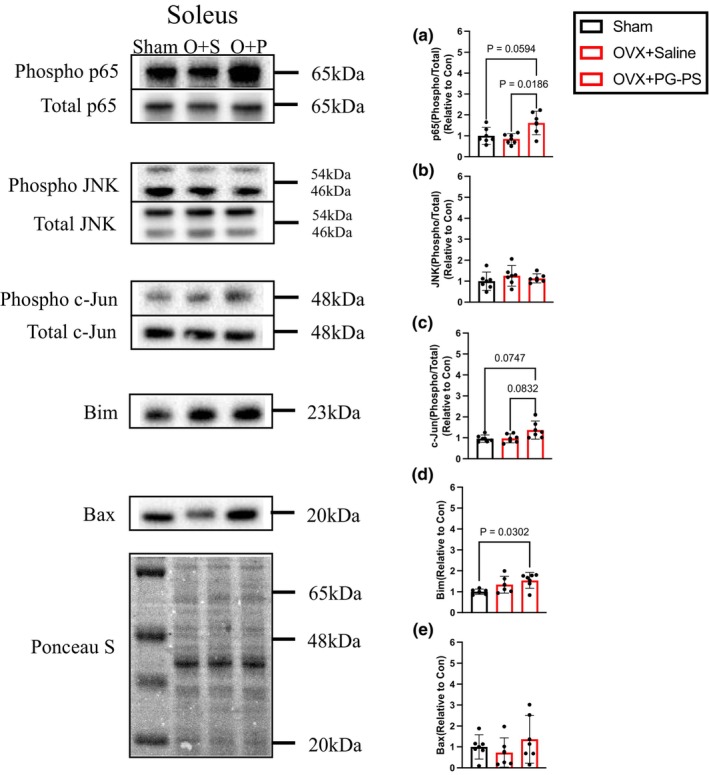
Inflammation‐related proteins and MPS in female mice. (a) Phosphorylated per total p65 of soleus muscle in female mice (Sham: *n* = 6, OVX + Saline; *n* = 6, OVX + PG‐PS: *n* = 6). (b) Phosphorylated per total JNK of soleus muscle in female mice (Sham: *n* = 6, OVX + Saline; *n* = 6, OVX + PG‐PS: *n* = 6). (c) Phosphorylated per total c‐Jun of soleus muscle in female mice (Sham: *n* = 6, OVX + Saline; *n* = 6, OVX + PG‐PS: *n* = 6). (d) Expression of Bim in female mice soleus muscle (Sham: *n* = 6, OVX + Saline; *n* = 6, OVX + PG‐PS: *n* = 6). (e) Expression of Bax in female mice soleus muscle (Sham: *n* = 6, OVX + Saline; *n* = 6, OVX + PG‐PS: *n* = 6). Data are shown as the mean ± SD. One‐way analysis of variance (ANOVA) was performed, followed by a post hoc test.

We analyzed whether the same protein activation occurs in OVX and male mice. The OVX + PG‐PS group exhibited significantly increased Bim expression compared to the sham group. JNK phosphorylation and Bax expression did not significantly differ among the groups. In contrast, PG‐PS administration increased phosphorylated c‐Jun levels (*p* = 0.083). Moreover, phosphorylated p65 levels were significantly higher in the OVX + PG‐PS group than in the OVX + Saline group (*p* = 0.018, Figure 11).

### 3.11 PG‐PS Administration increased MPS in OVX mice

**FIGURE 12:**
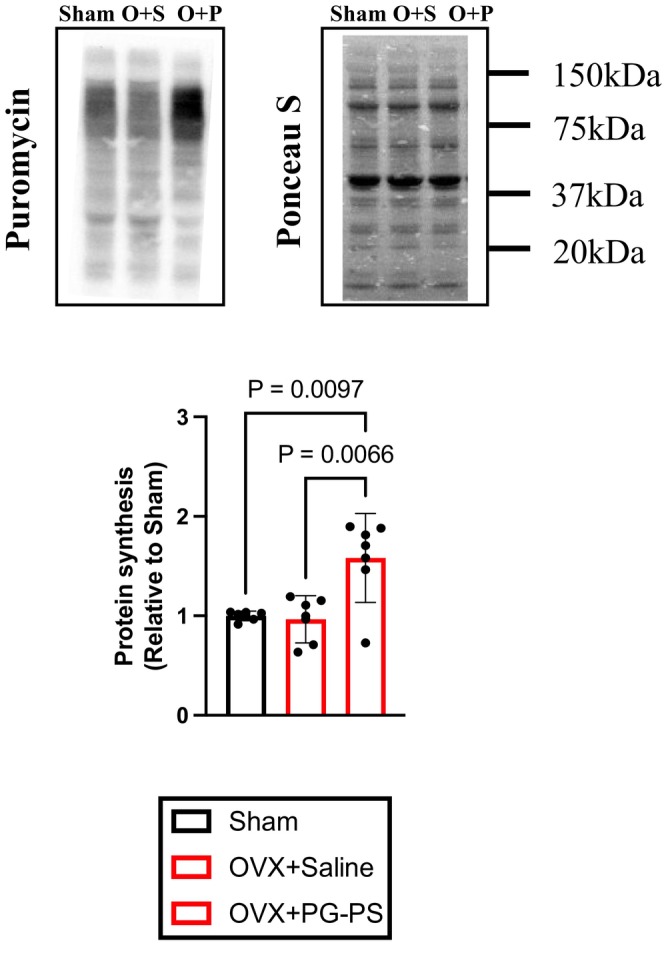
MPS in OVX mice. Puromycin‐incorporated protein and Ponceau S of gastrocnemius muscle in female mice. (Sham: *N* = 6, OVX + Saline; *n* = 6, OVX + PG‐PS: *n* = 6). Data are shown as the mean ± SD. One‐way analysis of variance (ANOVA) was performed, followed by a post hoc test.

Next, we examined the impact of PG‐PS on MPS in OVX mice. MPS levels were similar between the sham and OVX + Saline groups. However, contrary to expectations, the OVX + PG‐PS group exhibited significantly increased MPS compared to the sham and OVX + Saline groups (*p* = 0.009 and *p* = 0.006, respectively, Figure 12).

## 4 DISCUSSION

In this study, we used a PG‐PS‐induced CSI model mice to investigate whether skeletal muscle atrophy caused by CSI differs between sexes. The main findings were: (1) PG‐PS induced CSI in both sexes; however, skeletal muscle atrophy was observed only in male mice, suggesting sex‐specific differences. (2) Male mice exhibited skeletal muscle atrophy associated with reduced baseline MPS and increased apoptosis. (3) In OVX mice with PG‐PS‐induced skeletal muscle atrophy, ovarian functions, including hormone secretion, appeared to influence sex differences in CSI‐induced skeletal muscle atrophy. However, the mechanisms underlying atrophy in male mice seemed distinct.

### 4.1 Sex differences in CSI‐induced skeletal muscle atrophy

A previous study showed that PG‐PS induces skeletal muscle atrophy in female Lewis rats (Sumi et al., 2020a). In this study, C57BL6J mice were used because of the availability of the study population and ease of intervention. Unlike Lewis rats, skeletal muscle atrophy was not observed in female C57BL/6J mice, likely owing to strain differences. C57BL/6J mice generally exhibit a more robust inflammatory response during the acute phase than other strains (Fulton et al., 2006; Steven et al., 2017). This suggested that the differing immune systems of mice and rats may have resulted in variations in the manifestation of muscle atrophy.

PG‐PS treatment has been shown to increase the plasma levels of TNF‐α and IL‐1β in rats, suggesting that these circulating inflammatory cytokines are involved in skeletal muscle loss and muscle weakness (Sumi et al., 2020b). Additionally, Kimpel et al. (2003) reported that splenectomy attenuates the inflammatory response induced by PG‐PS, suggesting that PG‐PS treatment induces systemic inflammation in the spleen. Therefore, we investigated those protein levels and spleen weight in the blood plasma of the mice 3 weeks after the PG‐PS injection. In the present study, an increase in cytokine concentrations and spleen weight was also observed following PG‐PS treatment.

Skeletal muscle mass is regulated by the balance between MPS and MPB. Previous studies have demonstrated that inflammatory cytokines and ROS suppress MPS (Chen et al., 2023). CSI is also known to attenuate protein synthesis in skeletal muscles (Rudrappa et al., 2016; Sumi et al., 2020b). Therefore, we measured the MPS levels using puromycin. However, Goodman et al. (2011) and Yoshimura and Nomura (2022) reported that the quantity and quality of food affect MPS, as estimated using puromycin labelling. In this study, male PG‐PS‐treated mice slightly decreased food intake on Day 20. Therefore, we needed to measure MPS under conditions that excluded the effects of food intake. As a result, a decrease in MPS was observed only in male mice.

PG‐PS selectively decreased fiber size in the soleus muscle. A previous study reported a negative correlation between skeletal muscle weight and circulating TNF‐α levels in PG‐PS‐treated Lewis rats (Sumi et al., 2020b), suggesting that TNF‐α decreased muscle weight. Tang et al. (2013) demonstrated that TNF‐α overexpression in transgenic male mice (background: C57BL/6 mice) specifically caused soleus muscle atrophy. Conversely, they observed no skeletal muscle atrophy in female mice. TNF‐α activates the JNK signaling pathway (Bax and c‐Jun etc.) through ER stress, leading to the induction of apoptosis (Draijer et al., 2016; Sinha‐Hikim et al., 2007). Additionally, ER stress induces apoptosis and inhibits protein translation in skeletal muscles (Afroze & Kumar, 2019; Dirks & Leeuwenburgh, 2005). This suggests that TNF‐α‐induced ER stress caused increased apoptosis and decreased MPS in male mice. In contrast, female mice showed no significant differences in apoptosis or MPS. Previous studies have reported that sex hormones, such as estrogen, mitigate intracellular stress signaling pathways and excessive Ca^2+^ influx into the cytoplasm (Michelucci et al., 2017). In this study, sex differences in skeletal muscle atrophy may have been caused by sex hormones suppressing the TNF‐α signaling pathway and intracellular stress. On the other hand, several reports have indicated that bacterial components can directly induce muscle atrophy (Ducharme et al., 2023; Wang et al., 2023). Therefore, future research should focus on elucidating the detailed molecular mechanisms underlying these sex‐specific differences in CSI‐induced skeletal muscle atrophy.

### 4.2 CSI in OVX mice induced skeletal muscle atrophy

Administration of PG‐PS to both male and female mice resulted in increased spleen weight and the induction of CSI. PG‐PS‐induced CSI has been shown to be mediated via the spleen, an immune‐related organ (Kimpel et al., 2003). The observation of splenomegaly in the OVX model suggests that CSI was also appropriately induced under the experimental conditions using OVX mice.

The OVX + Saline group showed an increase in skeletal muscle weight. However, no significant change was observed in the fiber size. Ono et al. reported that OVX mice had temporarily increased skeletal muscle mass 8 weeks after surgery, although there was no change in CSA (Kitajima & Ono, 2016). This suggests that alterations in the interstitial connective tissue might have contributed to the increased muscle mass in OVX mice. In this study, dissection was performed 7 weeks after OVX. Therefore, increasing muscle mass after OVX may not result in substantial muscle hypertrophy. In contrast, the OVX + PG‐PS group showed decreased skeletal muscle weight and fiber size. Additionally, phosphorylation of c‐Jun and p65 was increased in atrophic muscle. Phosphorylation of p65 in skeletal muscle leads to an increase in ubiquitin‐related proteins (Perry et al., 2016). Ovaries are essential for female hormone production, and these hormones regulate the immune system (Gay et al., 2021; Groh et al., 2022; Straub, 2007). Additionally, sex hormones directly contribute to maintaining skeletal muscle protein homeostasis (Seko et al., 2020; Toth et al., 2001). Therefore, the absence of ovarian hormones may activate these complex interacting factors, thereby promoting both apoptosis and the ubiquitin‐proteasome pathway, which could lead to skeletal muscle atrophy.

In this study, MPS was measured using the SUnSET method. The OVX + Saline group did not exhibit any changes in MPS. However, PG‐PS administration induced skeletal muscle atrophy and significantly increased MPS. The SUnSET method quantifies puromycin incorporation into the nascent peptide chain (Penman et al., 1964; Schmidt et al., 2009). Puromycin suppresses protein synthesis by blocking translation, suggesting that it suppressed the synthesis of MPB‐related, skeletal muscle structural, and MPS‐related proteins. Therefore, increased levels of puromycin‐incorporated proteins in OVX + PG‐PS mice may indicate the over‐translation of MPB‐related proteins. Future studies should analyze MPB‐related proteins to determine whether skeletal muscle mass can be maintained when this factor is suppressed.

Inflammation‐induced skeletal muscle atrophy differs by sex (Tang et al., 2013; Zhong et al., 2022). Generally, males have more severe skeletal muscle atrophy than females. This study also revealed that PG‐PS‐induced CSI led to sex‐dependent skeletal muscle atrophy. In addition, skeletal muscle atrophy induced by PG‐PS was also observed in OVX mice. Therefore, it is suggested that the sex differences in skeletal muscle atrophy induced by CSI may be regulated by the function of the gonads, including the production of sex hormones such as estrogen and progesterone. The significance of this study lies in providing insights into the molecular mechanisms underlying the process of CSI‐induced skeletal muscle atrophy due to aging. Additionally, these findings contribute to understanding the initial characteristics of the PG‐PS model. Future studies should investigate hormone replacement therapy to mitigate atrophy and the molecular mechanisms of PG‐PS‐induced skeletal muscle atrophy in orchiectomy mice.

## 5 LIMITATIONS

This study has some limitations. First, we did not identify the upstream factors associated with MPS or MPB. Second, the lack of a Sham + PG‐PS group in the OVX experiment is a limitation. Third, although we examined the mechanism underlying the increased MPS in the OVX + PG‐PS group, further investigation is needed. Finally, all data were obtained 3 weeks after PG‐PS administration; therefore, we could not assess the molecular dynamics at various time points. Future studies should examine time‐dependent changes to deepen our understanding of sex differences in CSI‐induced skeletal muscle atrophy.

We investigated whether CSI‐induced skeletal muscle atrophy differs between sexes in PG‐PS model mice. PG‐PS induced CSI in both sexes. However, skeletal muscle atrophy was observed only in male mice. This atrophy was associated with increased apoptosis and decreased MPS. Additionally, OVX mice exhibited decreased skeletal muscle weight and fiber size after PG‐PS administration. These findings suggest that sex differences in CSI‐induced skeletal muscle atrophy are related to gonadal function, including sex hormone production.

## AUTHOR CONTRIBUTIONS

R.K. and K.N. conceived and designed the study. R.K., J.J., S.H., T.Y., T.K., K.K., Y.K., and K.N. performed the experiments. R.K. analyzed the data. R.K., J.J., S.H., T.Y., T.K., K.K., Y.K., and K.N. interpreted the results of the experiments. R.K. prepared figures. R.K. and K.N. drafted the manuscript. R.K., J.J., S.H., T.Y., T.K., K.K., Y.K., and K.N. edited and revised the manuscript. R.K., J.J., S.H., T.Y., T.K., K.K., Y.K., and K.N. approved the final version of this manuscript.

## FUNDING INFORMATION

This project was funded through institutional support from the Nippon Sport Science University.

## CONFLICT OF INTEREST STATEMENT

The authors declare no conflicts of interest, financial or otherwise.

## Data Availability

All data generated and analyzed in the present study are available from the corresponding author on reasonable request.
